# The Treatment of Criminals

**Published:** 1896-02-08

**Authors:** 


### Modern Sociology.

### THE TREATMENT OF CRIMINALS.

In the treatment of criminals three main principles must always be kept in view, prevention, repression, and reformation, and although it is not for a moment to be supposed that perfection has anywhere been attained, there is probably no country in the world, according to Mr. Tallack,* which has so successfully sought to secure this tri-unity as Great Britain. For examples of the extremes to which people may go both in cruel repression and in such mildness of discipline as ceases to deter from crime we must look abroad. To those who look upon Russia as a civilised country, it must be a startling thing to hear that, such is the savagery with which criminals, even of small degree, are there dealt with, in 1893 it was possible to present statistics to the Czar showing that in the preceding ten years three thousand persons, mostly guilty merely of agricultural thefts, had died from the effects of the cruel punishment of the knout—flogged to death. The charges made against Russian prisons are well known, and are often disbelieved, but so lately as 1894 Prince Nicholas Galitzen, who had been sent out with full powers from the Czar to visit and examine thoroughly the Siberian penal settlements, said in his private report, "The only reform good enough for some of these pest-holes is a can of petroleum and a few matches," adding that Mr. Kennan did not exaggerate in his account of some of these prisons.

Turning to another part of the world, there is a great prison in Santiago, in Chili, in one part of which the inmates are employed usefully and under humane conditions, yet in another part of this "model" prison there are noisome, slimy cells, into which daylight never enters, and in which human beings are literally buried alive. A traveller (F. B. Ward) thus describes matters in 1893: "Under the massive arches. . . . are inner cells, two feet wide by six feet long, and destitute of a single article of furniture. Until recently, those confined in them were walled in, the bricks being cemented in place over the living tomb. Now there is a thick iron door, which is securely nailed up and fastened all round with huge clamps, . . . . and over all the great red seal of the Government is placed —not to be removed until the man is dead, or his sentence has expired. . . . The prisoners cannot tell night from day. . . . Low down in the iron door, close to the ground, is a tiny sliding panel, a foot long by a few inches wide, arranged like a double drawer, so that food and water may be slipped in on shallow pans and the refuse removed. Twice in every twenty-four hours this panel is operated, and if the food remains untouched a given number of days, it is known to a certainty that the man is dead, and only then can the door be unsealed unless his time is up. . . . In the same clothes he wears on entering, unwashed, uncombed, without even a blanket or a handful of straw to lie upon, he languishes in sickness, lives or dies, with no means of making his condition known to those outside. He may count the lagging hours, sleep, rave, curse, pray, long for death, dash his brains out, go mad if he likes—nobody knows it. He is dead to the world and buried, though living."

* Tallack. Penological and preventive principles.

In the United States the great curse of the prison system is just the opposite to this, arising mainly from the association of the prisoners. "Thieves, tramps, and other offenders enjoy the dirty, lazy life in many of these gaols, because they have fire, good food, cards, tobacco, and whisky. . . . But to the more respectable or still unhardened prisoner such places and the horrible conversation are means of torture."

In some of the Southern States the practice of "farming out" the convicts to contractors, for labour in the open air, seems still to prevail. Even little children of both sexes are committed to this virtual slavery for protracted terms. In these establishments very inadequate attention is paid to the separation of the sexes, and gross immorality is frequent, many wretched infants being born in them.

A New York journal, describing the horrors of the Southern States convict camps, says truly enough: "It is barbarous to confine women in the same prison-pens with a horde of desperate ruffians, who respect nothing under heaven."

In certain of the prisons in the Northern States the opposite evil of extreme laxity, combined with the error of allowing association among the prisoners, is carried to such an extent that we find prisoners saying, "Yes, ma'am, I'm very comfortable here; quite contented here"; whereas it certainly is altogether contrary to penological principles that any criminal should be contented with prison life. There is a model prison at Elmira in which the advantages extended to prisoners are such as to excite the envy of honest toilers outside. The "collegiate training" of the inmates is a prominent feature, teachers and professors being engaged to instruct classes and deliver lectures on various topics, including writing, drawing, designing, German, English, and American history, business law, arithmetic, physical geography, economics, practical ethics, political science, &c.

A paper written by a prisoner in Elmira on a cold snowy day compassionately alludes to the wretched homes, almost visible from the prison walls, where ill-clad and ill-fed children and wives of unemployed or weary men were crouching in the cold, and contrasts their lot with that of the convicts, adding, "Here, at this prison, 'tis the dinner hour; up from the great dining-hall below rises the fragrant odour of good food and the hum of animated voices, with rippling laughter interspersed. The food is hot, and sufficient as to quantity; the apartments are warmed with steam, and after the short day is past, the electric light brightens things for the long evenings—long, but not dreary, for books are abundant." It should be noted that this prison is not for selected good-class prisoners only, for it receives them of every grade, "murderers, burglars, thieves, and violators of women."

Cruel to the individual as are the worst forms of "solitary confinement," it may be questioned whether such attractive forms of associated imprisonment as are here described, are not, by their lack of repressive influence, even more cruel to the community.

